# Clinical profile and 90 day outcomes of 10 851 heart failure patients across India: National Heart Failure Registry

**DOI:** 10.1002/ehf2.14096

**Published:** 2022-08-10

**Authors:** Sivadasanpillai Harikrishnan, Ajay Bahl, Ambuj Roy, Animesh Mishra, Jayesh Prajapati, C.N. Manjunath, Rishi Sethi, Santanu Guha, Santhosh Satheesh, R.S. Dhaliwal, Meenakshi Sarma, Sanjay Ganapathy, Panniyammakal Jeemon, for the NHFR investigators, Jabir Abdullakutty, Stigi Joseph, Sajan Narayanan, Rajesh G, Anwar C. Varghese, Ramakrishna Damodara, Johny Joseph, Deepak Davidson, Joby K. Thomas, Thomas George, Shafeeq Mattummal, Nitish Naik, Sandeep Singh, Gautam Sharma, Sandeep Seth, Girish Palleda, Mohit D. Gupta, Prabhat Kumar, Nirav Kumar, Malani Susheel, Major Vijay Vohra, Prakash C. Negi, Sanjeev Asotra, Kunal Mahajan, Rajesh Sharma, Balaraju D, Sathwik Raj, Anand Katageri, Veena Nanjappa, Ranjan Shetty, Rockey Katheria, Maneesh Rai, Muhammed Musthafa M, Subrahmanyam DKS, Raja Selvaraj, Vivekanandan M, Vindhya RJ, Durgaprasad Rajasekhar, Vanajakashamma V, K. Sreedhar Naik, Justin Paul Gnanaraj, Fazil Hussain, Swaminathan N, Sooraj Menon, Hemanath TR, Selvarani G, Balasubramanian S, Veeramani SR, Anoop George Alex, Soundarya G, Sreekanth Yerram, Srinivas Bhyravavajhala, Jyotsna Maddury, Sai Satish Oruganti, Saurabh Mehrotra, Neelam Dahiya, Vibhuti Sharma, Ambika Sood, Bishav Mohan, Rohit Tandon, Col Navreet Singh, Inderjeet Monga, Jeet Ram Kashyap, Sreenivas Reddy, Mukul Kumar, Daljeet Guleria, Anurag Sharma, Roopesh Singhal, Hasit Joshi, Mary Iby, Bhavesh Roy, Parth Thakkar, Dinesh Choudhary, Devendra Kumar Agarwal, Ajay Swamy, Monga IC, Shomu Bohora, Akshayaya Pradhan, Pravesh Vishwakarma, Aditya Kapoor, Sudeep Kumar, Dharmendra Jain, Umesh Pande, Suyash Tripathi, Bhupendra Verma, Soumik Ghosh, Rajpal Prajapati, Krishna Santosh Vemuri, Abhishek Kaushley, Suraj Chaturvedi, Nikhil Jha, Sushil Kumar, Ankit Krishna Agrawal, Narendra Kumar, Sandeep Chowdhary, Smit Shrivastava, B.S. Yadav, Rajeev Gupta, R.K. Singh, Gurumeet Singh, Prokash Chandra Bagchi, Tannu Kumari, Mukesh Kumar Agrawal, Manoranjan Mondal, Sankar C. Mandal, Kanak Kumar Mitra, S.N. Routray, Dipak Ranjan Das, Trinath Kumar Mishra, Amit Malviya, Adorelia Laitthma, Rinchin Dorjee, Hem Chandra Kalita, Mriganka Shekhar Chaliha, Dibya Jyothi Dutta, Nisar Ahmad Tramboo, Aamir Rashid, Ravinder Singh Rao, Hemant Chaturvedi, Guruprasad D. Naik, Ramnath Nevrekar

**Affiliations:** ^1^ Cardiology Sree Chitra Tirunal Institute for Medical Sciences and Technology (SCTIMST) Trivandrum India; ^2^ Cardiology Postgraduate Institute of Medical Education and Research (PGIMER) Chandigarh India; ^3^ Cardiology All India Institute of Medical Sciences (AIIMS) New Delhi India; ^4^ Cardiology North Eastern Indira Gandhi Regional Institute of Health and Medical Sciences (NEIGRIHMS) Shillong India; ^5^ Cardiology UN Mehta Institute of Cardiology and Research Centre (UNMICRC) Ahmedabad India; ^6^ Cardiology Sri Jayadeva Institute of Cardiovascular Sciences and Research (SJICR) Bangalore India; ^7^ Cardiology King George's Medical University (KGMU) Lucknow India; ^8^ Cardiology Medical College Hospital (MCH) Kolkata India; ^9^ Cardiology Jawaharlal Institute of Postgraduate Medical Education and Research (JIPMER) Pondicherry India; ^10^ Division of Non‐Communicable Diseases Indian Council of Medical Research (ICMR) New Delhi India; ^11^ Achutha Menon Centre for Health Science Studies Sree Chitra Tirunal Institute for Medical Sciences and Technology (SCTIMST) Trivandrum 695011 Kerala India

**Keywords:** Guideline‐directed medical therapy, Heart failure, India, Mortality, National Heart Failure Registry

## Abstract

**Aims:**

Limited data on the uptake of guideline‐directed medical therapies (GDMTs) and the mortality of acute decompensated HF (ADHF) patients are available from India. The National Heart Failure Registry (NHFR) aimed to assess clinical presentation, practice patterns, and the mortality of ADHF patients in India.

**Methods and results:**

The NHFR is a facility‐based, multi‐centre clinical registry of consecutive ADHF patients with prospective follow‐up. Fifty three tertiary care hospitals in 21 states in India participated in the NHFR. All consecutive ADHF patients who satisfied the European Society of Cardiology criteria were enrolled in the registry. All‐cause mortality at 90 days was the main outcome measure. In total, 10 851 consecutive patients were recruited (mean age: 59.9 years, 31% women). Ischaemic heart disease was the predominant aetiology for HF (72%), followed by dilated cardiomyopathy (18%). Isolated right HF was noted in 62 (0.6%) participants. In eligible HF patients, 47.5% received GDMT. The 90 day mortality was 14.2% (14.9% and 13.9% in women and men, respectively) with a re‐admission rate of 8.4%. An inverse relationship between educational class based on years of education and 90 day mortality (high mortality in the lowest educational class) was observed in the study population. Patients with HF with reduced ejection fraction and HF with mildly reduced ejection fraction who did not receive GDMT experienced higher mortality (log‐rank *P* < 0.001) than those who received GDMT. Baseline educational class, body mass index, New York Heart Association functional class, ejection fraction, dependent oedema, serum creatinine, QRS > 120 ms, atrial fibrillation, mitral regurgitation, haemoglobin levels, serum sodium, and GDMT independently predicted 90 day mortality.

**Conclusion:**

One of seven ADHF patients in the NHFR died during the first 90 days of follow‐up. One of two patients received GDMT. Adherence to GDMT improved survival in HF patients with reduced and mildly reduced ejection fractions. Our findings call for innovative quality improvement initiatives to improve the uptake of GDMT among HF patients in India.

## Introduction

Heart failure, a complex clinical syndrome often arising from impairment of ventricular filling or ejection of blood, is estimated to affect 26 million people worldwide.^1^ It is disabling and deadly, with an annual hospitalization of approximately one million US adults^2^ and a 1 year mortality of 23.6% in patients hospitalized with acute HF in high‐income settings.^3^

Heart failure prevalence varies across regions of the world.^4^, ^5^ However, population‐based data on HF prevalence and incidence from India are scarce. Data from the Trivandrum Heart Failure Registry,^6^, ^7^, ^8^ the International Congestive Heart Failure registry,^9^ and the Medanta registry^10^ provide crucial information on patient characteristics, prevailing treatment practices, and survival of HF in India. However, all of these registries are limited in their geographical representations of India.^11^ The National Heart Failure Registry (NHFR) tries to bridge the gap with representation from diverse geographical areas of India. We describe the demographic characteristics, disease aetiology, practice patterns, 90 day mortality, and predictors of 90 day mortality of acute decompensated HF (ADHF) patients from the NHFR.

## Methods

### Study settings

The design and detailed methods of the NHFR have been published earlier.^12^ In brief, the NHFR is a facility‐based registry of ADHF patients admitted to 53 hospitals in 21 states and four union territories in India. The overall recruitment period was from January 2019 to July 2020. Nine nodal centres that were government‐funded medical institutes with adequate experience in conducting epidemiological studies were selected from nine different regions in India. Each nodal centre identified five additional hospitals, representing the different geographies and ethnicities in the region as participating sites. The 53 centres included in the NHFR ensured adequate representation of different geographical regions of India (see Supporting Information, *Figure* *S1*).

### Participants

All consecutive ADHF patients >18 years who satisfied the European Society of Cardiology (ESC) 2016 criteria^13^ were enrolled in the registry. All included patients had an echocardiogram during the hospitalization period.

### Data collection and definitions

Data on socio‐demographic characteristics, aetiology of HF, medical history, medications, clinical presentation, in‐hospital diagnosis, and treatment were retrieved from medical records and interviews of the patients or their immediate family members. Trained staff nurses collected relevant data and uploaded them to the NHFR central server using either an Android‐based or a web‐based data entry application. Data uploaded to the NHFR server were verified for accuracy and completeness by the nodal centre co‐ordinator. The data quality was monitored using centrally generated periodic listing of data queries. All related data queries were reviewed and resolved by the nodal centre co‐ordinators with the help of the National Coordinating Centre at Sree Chitra Tirunal Institute for Medical Sciences and Technology (SCTIMST), Trivandrum, India. Further, 10% of data fields were subjected to source data verification, and all participating sites reported <1% data errors.

We categorized HF into three groups based on the universal definition of HF^14^: HF with reduced ejection fraction (EF) or HFrEF (left ventricular EF based on echocardiogram < 40%), HF with mildly reduced EF or HFmrEF (EF 41–49%),^15^ and HF with preserved EF or HFpEF (EF > 50% and structural heart disease by echocardiogram).^16^ Guideline‐directed medical therapy (GDMT) was considered as a combination of angiotensin‐converting enzyme inhibitors (ACEIs)/angiotensin receptor blockers (ARBs)/angiotensin receptor–neprilysin inhibitors (ARNIs), beta‐blockers, and aldosterone blockers in HFrEF.

### Longitudinal follow‐up

The site co‐ordinators or research nurses at each site followed up all patients enrolled in the NHFR for a minimum period of 3 months from the date of admission. The follow‐up data were collected either during the clinic visits or by telephone. Data related to repeat hospitalizations, mortality, and cause of death were collected directly either from the patients or from their immediate family members. The date of such events was also collected and recorded as part of the study. In the case of out‐of‐hospital mortality, the research staff completed a verbal autopsy to ascertain the cause of death.

### Outcomes

All‐cause mortality at 90 days was the main outcome variable. We used the time‐to‐mortality data based on the date of event in the survival analysis.

### Statistical analysis

Categorical baseline variables are presented as proportions and compared across the three HF categories by using a chi square test. Distribution of continuous variables was checked, and normal distribution was ensured before applying any parametric hypothesis testing. Continuous variables are presented as means with standard deviation (SD). We used one‐way ANOVA for comparison of means across the three HF categories.

We reported in‐hospital, 90 day mortality rate as a proportion (number of deaths/total registered patients) and per 100 person‐days of follow‐up. Further, we employed survival analyses to identify factors associated with 90 day mortality outcomes. Kaplan–Meier survival models were performed initially, after checking the proportional hazards' assumption by using log‐minus‐log plots, and groups were compared using log‐rank tests. Finally, a multi‐variable Cox proportional hazard model (Cox‐PH) was employed to assess the adjusted hazard ratio (HR) of all‐cause mortality. We used variables that are associated with mortality in previous models of acute HF^17^, ^18^ and are available in the NHFR data set to generate the regression model. All analyses were carried out using Stata 16.1 StataCorp LLC.

### Ethical considerations

We obtained institutional ethics committee approval from all participating centres before the commencement of the study. The Institute Ethics Commitee of the co‐ordinating centre (SCTIMST) also cleared the study (no.: SCT/IEC/1167). We registered this study in the Clinical Trial Registry of India (CTRI/2019/01/017053). A written informed consent was obtained from all participants before enrolment into the registry.

### Funding

We received a research grant from the Indian Council of Medical Research (ICMR), Department of Health Research, Government of India, for the conduct of this study.

## Results

A total of 10 851 patients were recruited. The mean age of the study population was 59.9 (SD = 13.5) years (*Table* *1*). Women contributed to less than one‐third (31%) of the study population. The mean year of schooling was 7.8 (SD = 5.2) years. One‐fourth of the study population (25.5%) reported less than 4 years of formal schooling.

**Table 1:** Baseline characteristics of the study population

Variables	Overall (*N* = 10 851)	HFrEF (*N* = 7082)	HFmrEF (*N* = 2396)	HFpEF (*N* = 1373)	*P*‐value
Age in years, mean (SD)	59.9 (13.5)	60.2 (13.2)	59.9 (13.0)	58.8 (15.7)	0.002
Women, *n* (%)	3366 (31.0)	1975 (27.9)	735 (30.7)	656 (47.8)	<0.001
BMI in kg/m^2^, mean (SD)	24.2 (4.0)	24.3 (4.0)	24.2 (4.0)	23.9 (4.2)	0.068
Regions, *n* (%)					<0.001
Low ETLs	2425 (22.4)	1490 (21.0)	686 (28.6)	249 (18.1)	
Lower‐middle ETLs	1101 (10.2)	828 (11.7)	188 (7.9)	85 (6.2)	
Higher‐middle ETLs	4169 (38.4)	2863 (40.4)	808 (33.7)	498 (36.3)	
High ETLs	3156 (29.1)	1901 (26.8)	714 (29.8)	541 (39.4)	
Educational status, *n* (%)					<0.001
≤4 years of schooling	2765 (25.5)	1856 (26.2)	556 (23.2)	353 (25.7)	
5–8 years of schooling	2804 (25.9)	1821 (25.7)	619 (25.9)	364 (26.5)	
9–12 years of schooling	3815 (35.2)	2395 (33.9)	916 (38.3)	504 (36.7)	
>13 years of schooling	1459 (13.5)	1003 (14.2)	304 (12.7)	152 (11.1)	
Disease aetiology, *n* (%)					
Ischaemic heart disease	7801 (71.9)	5078 (71.7)	2047 (85.4)	676 (49.2)	<0.001
Rheumatic heart disease	641 (5.9)	179 (2.5)	128 (5.3)	334 (24.3)	<0.001
Non‐rheumatic VHD	231 (2.1)	123 (1.7)	28 (1.2)	80 (5.8)	<0.001
Dilated cardiomyopathy	1955 (18.0)	1772 (25.0)	160 (6.7)	23 (1.7)	<0.001
Hypertrophic cardiomyopathy	118 (1.1)	41 (0.6)	14 (0.6)	63 (4.6)	<0.001
Restrictive cardiomyopathy	40 (0.4)	11 (0.2)	7 (0.3)	22 (1.6)	<0.001
Peripartum cardiomyopathy	51 (0.5)	41 (0.6)	8 (0.3)	2 (0.2)	0.054
Congenital heart disease	124 (1.1)	39 (0.6)	39 (1.6)	46 (3.4)	<0.001
Myocarditis	58 (0.5)	29 (0.4)	23 (1.0)	6 (0.4)	0.005
Infective endocarditis	20 (0.2)	5 (0.1)	2 (0.1)	13 (1.0)	<0.001
Habits, *n* (%)					
Current tobacco use	3670 (33.8)	2530 (35.7)	807 (33.7)	333 (24.3)	<0.001
Current alcohol use	1808 (16.7)	1207 (17.0)	434 (18.1)	167 (12.2)	<0.001
Co‐morbid conditions, *n* (%)					
Hypertension	5303 (48.9)	3318 (46.7)	1245 (52.0)	740 (53.9)	<0.001
Diabetes mellitus	4617 (42.3)	3031 (42.8)	1075 (44.9)	511 (37.2)	<0.001
Stroke	323 (3.0)	205 (2.9)	70 (2.9)	48 (3.5)	0.479
COPD	743 (6.9)	487 (6.9)	137 (5.7)	119 (8.9)	0.003
Chronic kidney disease	921 (8.5)	605 (8.5)	183 (7.6)	133 (9.7)	0.091
History of chemotherapy	62 (0.6)	42 (0.6)	11 (0.5)	9 (0.7)	0.683
Atrial fibrillation/flutter	1029 (9.5)	557 (7.9)	177 (7.4)	295 (21.5)	<0.001
Severe mitral regurgitation	953 (8.8)	707 (10.0)	119 (5.0)	127 (9.3)	<0.001
Hypothyroidism	492 (4.5)	308 (4.4)	93 (3.9)	91 (6.6)	<0.001
Hyperthyroidism	117 (1.1)	72 (1.0)	21 (0.9)	24 (1.8)	0.031
Severity of heart failure, *n* (%)					
Previous admission for HF, *n* (%)	2780 (25.6)	1944 (27.5)	497 (20.8)	339 (24.7)	<0.001
NYHA Class III or more, *n* (%)	7720 (71.2)	5335 (75.4)	1409 (58.9)	976 (71.1)	<0.001
Dependant oedema, *n* (%)	1258 (11.6)	871 (12.3)	186 (7.8)	201 (14.6)	<0.001
Clinical measurements					
Heart rate > 100 beats/min, *n* (%)	3789 (35.0)	2662 (37.6)	652 (27.3)	475 (34.6)	<0.001
Systolic BP < 90 mmHg, *n* (%)	500 (4.6)	356 (5.0)	85 (3.6)	59 (4.3)	0.01
Diastolic BP < 60 mmHg, *n* (%)	615 (5.7)	414 (5.9)	121 (5.1)	80 (5.8)	0.338
QRS duration > 120 ms, *n* (%)	2866 (26.4)	2178 (30.8)	428 (17.9)	260 (18.9)	<0.001
Haemoglobin in mg/dL, mean (SD)	12.1 (2.2)	12.2 (2.1)	12.1 (2.2)	11.5 (2.3)	<0.001
Serum creatinine in mg/dL, mean (SD)	1.5 (1.1)	1.5 (1.1)	1.4 (1.0)	1.5 (1.1)	<0.001
Serum sodium < 125 mg/dL, *n* (%)	354 (3.3)	229 (3.2)	75 (3.1)	50 (3.6)	0.685
Serum potassium > 5.5 mEq/L, *n* (%)	518 (4.5)	345 (4.9)	103 (4.3)	70 (5.1)	0.443

BMI, body mass index; BP, blood pressure; COPD, chronic obstructive pulmonary disease; ETL, epidemiological transition level of states; HF, heart failure; HFmrEF, heart failure with mildly reduced ejection fraction; HFpEF, heart failure with preserved ejection fraction; HFrEF, heart failure with reduced ejection fraction; NYHA, New York Heart Association; SD, standard deviation; VHD, valvular heart diseases.

The mean body mass index (BMI) of the study population was 24.2 (SD = 4.0) kg/m^2^. More than three‐fourths (74.4%) of the patients had admission with *de novo* HF, whereas the remaining (25.6%) patients had re‐admissions. The most common presentation was HFrEF in two‐thirds (65.2%) of the study population, followed by HFmrEF (22%) and HFpEF (12.7%). Ischaemic heart disease was the predominant aetiology for HF (72%), followed by dilated cardiomyopathy (18%). Rheumatic valvular heart disease (RHD) was present in 5.9% of the study population. Valve diseases other than RHD contributed 2.1% of the cases. Isolated right HF was noted in 62 (0.6%) participants.

Hypertension and diabetes were the most frequent co‐morbid conditions (48.9% and 42.3%, respectively). Atrial arrhythmia was prevalent in 9.5% of the study population. Chronic kidney disease, stroke, and chronic obstructive pulmonary disease (COPD) were reported in 8.5%, 3.0%, and 6.9% of the study population, respectively. Similarly, tobacco and alcohol use were reported in 33.8% and 16.7% of the study population, respectively.

More than two‐thirds (71.2%) of the patients presented with New York Heart Association (NYHA) Class III or Class IV. Further, about one‐third (35%) reported a baseline heart rate of more than 100 beats/min. A baseline systolic blood pressure of <90 mmHg and a diastolic blood pressure of <60 mmHg were reported in 4.6% and 5.7% of the study population, respectively. The mean serum creatinine was 1.5 (SD = 1.1) mg/dL, whereas the mean haemoglobin was 12.1 (SD = 2.2) g/dL. Serum sodium < 125 mEq/L and serum potassium > 5.5 mEq/L were reported in 3.3% and 4.5% of the study population, respectively.

### Prescription pattern and outcomes

**Figure 1:**
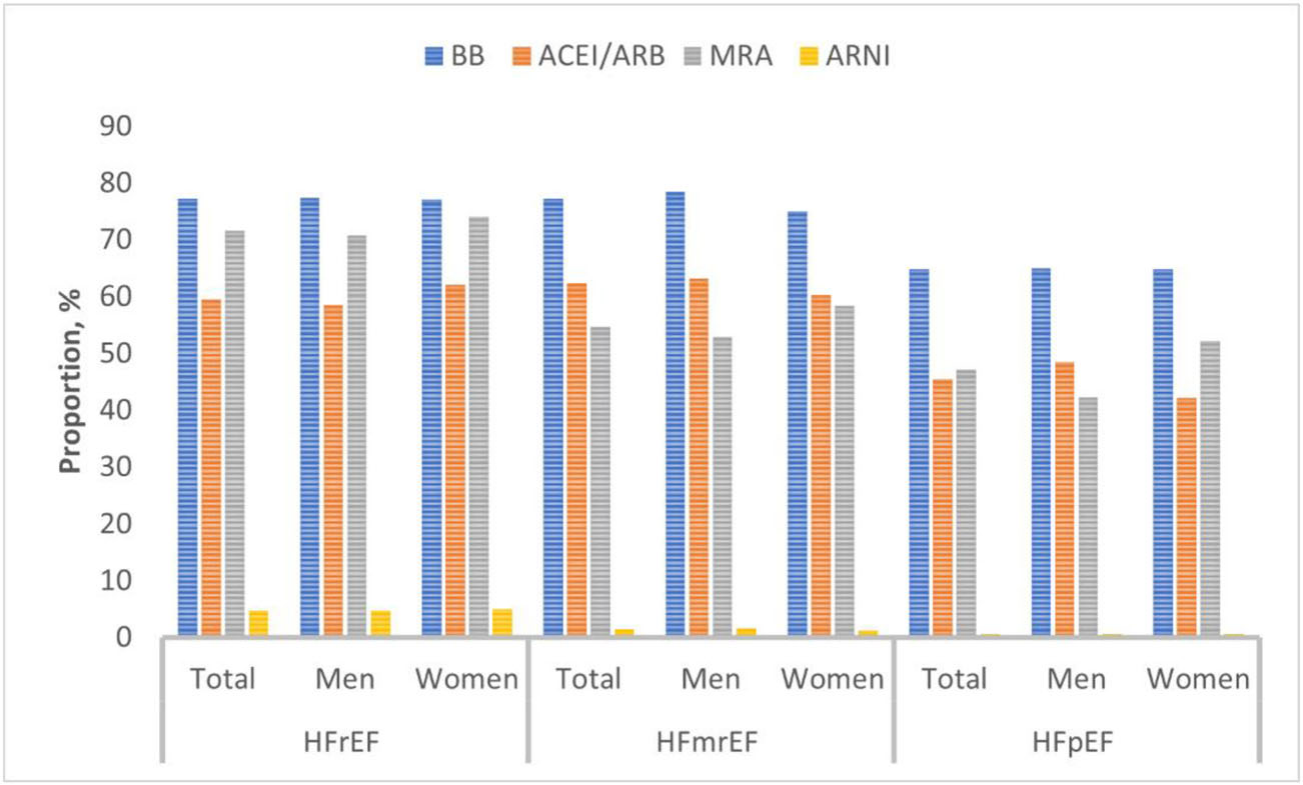
Intake of key drugs in men and women.ACEI, angiotensin‐converting enzyme inhibitor; ARB, angiotensin receptor blocker; ARNI, angiotensin receptor–neprilysin inhibitor; BB, beta‐blocker; HFmrEF, heart failure with mildly reduced ejection fraction; HFpEF, heart failure with preserved ejection fraction; HFrEF, heart failure with reduced ejection fraction; MRA, mineralocorticoid receptor antagonists.

During hospital admission, non‐invasive and invasive ventilatory support were required in 17.6% and 3.9% of the study population, respectively. Intravenous inotropes were administered in one‐fifth (20.2%) of the patient population. In HFrEF, 47.5% received GDMT (*Figure* *1*). Similarly, more than one‐third of HFmrEF also received GDMT. The ARNI use was very low in HFrEF (4.8%) and HFmrEF (4.7%) patients.

Digoxin was prescribed in 17.3%, 8.2%, and 16.9% of HFrEF, HFmrEF, and HFpEF patients, respectively (*Table* *2*). Ivabradine was prescribed in 15.1%, 7.8%, and 5.0% of the respective patient groups. The device use [cardiac resynchronization therapy (CRT) and implantable cardioverter defibrillators (ICD)] was very low (1.9%) in the study population. More than one‐third (35.9%) of the patients underwent coronary angiography (CAG), and half of them underwent percutaneous coronary intervention (PCI) (18.5%). Nearly 3.0% of patients were advised to undergo coronary artery bypass graft surgery (CABG).

**Table 2:** Pharmacologic therapy other than disease‐modifying agents

Drugs	HFrEF, *n* (%)			HFmrEF, *n* (%)			HFpEF, *n* (%)		
	Total	Men	Women	Total	Men	Women	Total	Men	Women
Digoxin	1225 (17.3)	831 (16.3)	394 (20.0)	197 (8.2)	103 (6.2)	94 (12.8)	232 (16.9)	94 (13.1)	138 (21.0)
Nitrates	1814 (25.6)	1350 (26.4)	464 (23.5)	776 (32.4)	550 (33.1)	226 (30.8)	267 (19.5)	168 (23.4)	99 (15.1)
Vasodilators	482 (6.8)	359 (7.0)	123 (6.2)	202 (8.4)	152 (9.2)	50 (6.8)	72 (5.2)	42 (5.9)	30 (4.6)
CCB	323 (4.6)	233 (4.6)	90 (4.6)	275 (11.5)	196 (11.8)	79 (10.8)	237 (17.3)	123 (17.2)	114 (17.4)
Heparin/LMWH	2542 (35.9)	1858 (36.4)	684 (34.6)	1088 (45.4)	765 (46.1)	323 (44.0)	502 (36.6)	264 (36.8)	238 (36.3)
OAC	363 (5.1)	257 (5.0)	106 (5.4)	108 (4.5)	55 (3.1)	53 (7.2)	230 (16.8)	104 (14.5)	126 (19.2)
Ivabradine	1070 (15.1)	767 (15.0)	303 (15.3)	187 (7.8)	112 (6.7)	75 (10.2)	68 (5.0)	39 (5.4)	29 (4.4)
Pulmonary vasodilators	219 (3.1)	174 (3.4)	45 (2.3)	119 (5.0)	84 (5.1)	35 (4.8)	70 (5.1)	38 (5.3)	32 (4.9)
Antiplatelets	4947 (69.9)	3682 (72.1)	1265 (64.1)	1871 (78.1)	1338 (80.1)	533 (72.5)	744 (54.2)	432 (60.3)	312 (47.6)
Inotropic agents	1712 (24.2)	1248 (24.4)	464 (23.5)	337 (14.1)	210 (12.6)	127 (17.3)	138 (10.1)	59 (8.2)	79 (12.0)

CCB, calcium channel blocker; HF, heart failure; HFmrEF, heart failure with mildly reduced ejection fraction; HFpEF, heart failure with preserved ejection fraction, HFrEF, heart failure with reduced ejection fraction; LMWH, low‐molecular‐weight heparin; OAC, oral anticoagulants.

**Figure 2:**
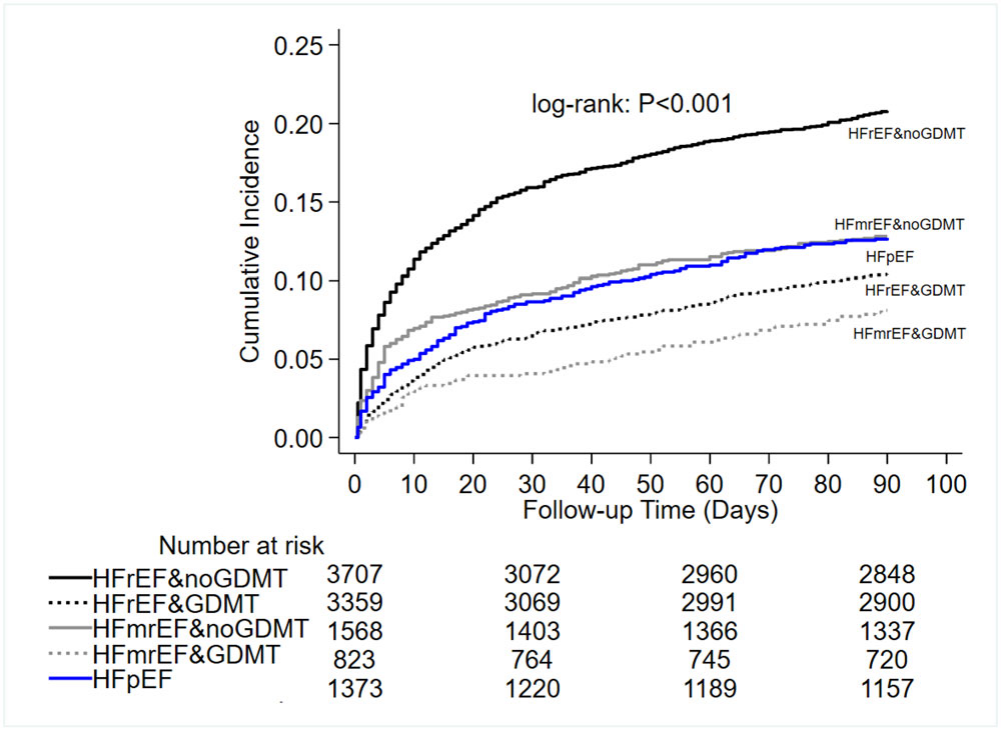
Guideline‐directed medical therapy, heart failure groups and 90 day survival.GDMT, guideline‐directed medical therapy; HFmrEF, heart failure with mildly reduced ejection fraction; HFpEF, heart failure with preserved ejection fraction; HFrEF, heart failure with reduced ejection fraction.

Overall, 90 day mortality was 14.2% (14.9% and 13.9% in women and men, respectively). In‐hospital mortality was 6.7% (*Table* *S1*). It was highest in HFrEF (7.5%) and lowest in HFmrEF (5.1%). In‐hospital mortality was 7.6% and 6.7% in women and men, respectively. Patients with HFrEF and HFmrEF who did not receive GDMT experienced higher mortality (log‐rank *P* < 0.001) than those who received it (*Figure* *2*). The 90 day mortality in HFmrEF was higher in women than in men (see Supporting Information, *Figure* *S2*). The re‐admission rate during the 90 day follow‐up was 8.4% in the study population.

**Figure 3:**
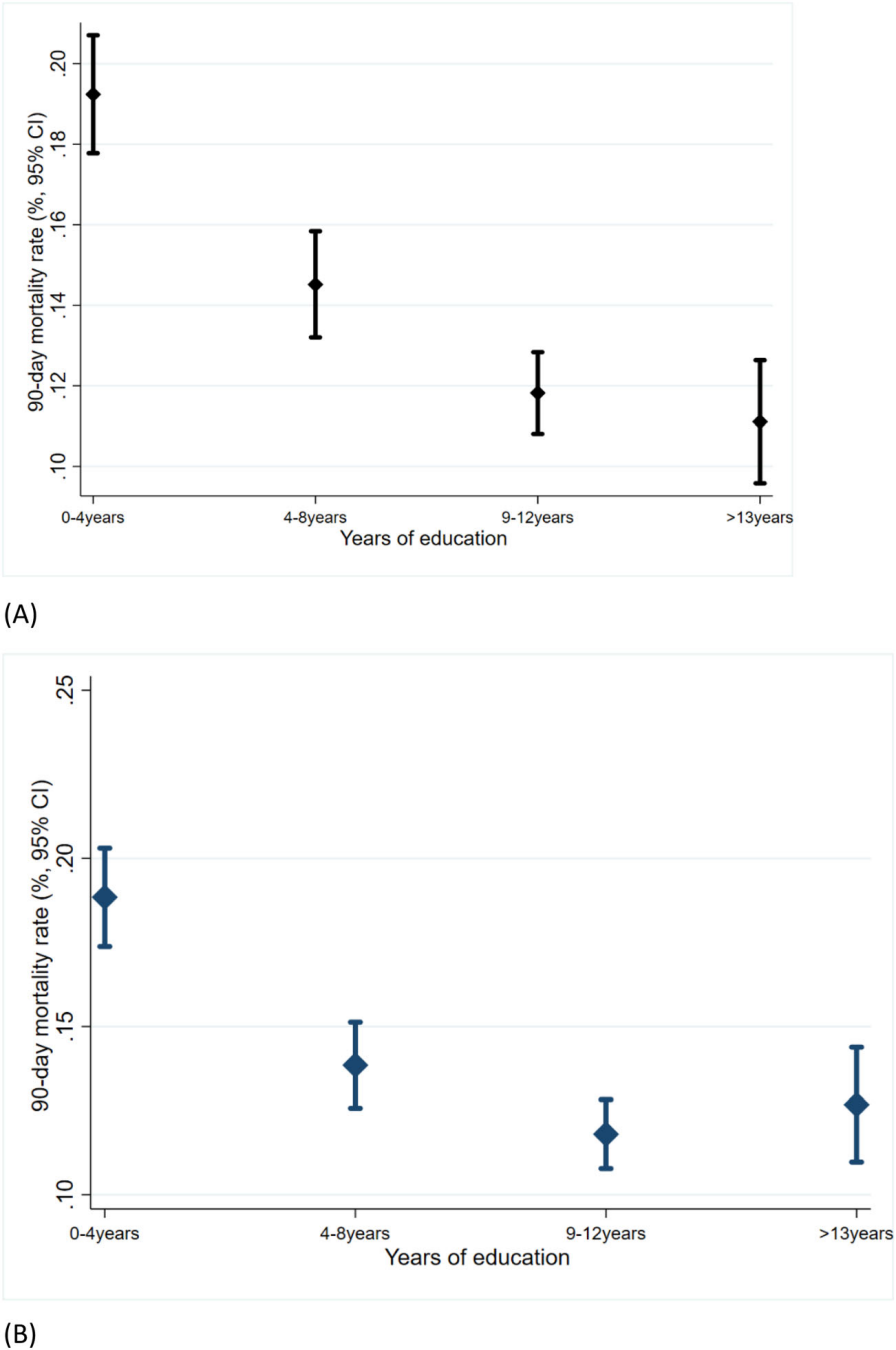
Educational status and mortality.(A) Crude mortality rate. (B) After adjustment for all variables as in the multi‐variable Cox proportional hazard model. CI, confidence interval.

An inverse relationship between educational class based on years of education and 90 day mortality (high mortality in the lowest educational class) was observed in the study population (*Figure* *3* *A*). The strength of the association did not attenuate in the multi‐variable Cox proportional hazard model (*Figure* *3* *B*). Further, baseline BMI, EF, NYHA class, dependant oedema, mitral regurgitation, QRS duration > 120 ms, presence of COPD, atrial fibrillation, use of GDMT, dilated cardiomyopathy, haemoglobin, serum sodium < 125 mEq/L, use of intravenous inotropes and diuretics, and respiratory support during admission were associated with 90 day mortality (*Table* *3*).

**Table 3:** Multi‐variable Cox proportional hazard models for 90 day mortality

Variables	HR (95% CI)^a^	*P*‐value
Age, 10 year increase	1.02 (0.98–1.07)	0.265
Gender		
Women	1	
Men	1.04 (0.93–1.17)	0.480
BMI, 1 kg/m^2^ increase	0.97 (0.96–0.98)	<0.001
NYHA class		
Class II	1	
Class III	1.36 (1.16–1.60)	<0.001
Class IV	1.61 (1.37–1.90)	<0.001
Ejection fraction		
HFrEF	1	
HFmrEF	0.95 (0.83–1.10)	0.509
HFpEF	0.77 (0.64–0.92)	0.004
Systolic blood pressure, mmHg	0.99 (0.98–0.99)	<0.001
Serum creatinine, mg/dL increase	1.09 (1.06–1.12)	<0.001
Tobacco use		
No	1	
Yes	0.94 (0.84–1.06)	0.323
Diabetes		
No	1	
Yes	0.95 (0.85–1.05)	0.316
Stroke		
No	1	
Yes	1.18 (0.93–1.51)	0.179
COPD		
No	1	
Yes	1.25 (1.05–1.49)	0.012
Previous admission for HF		
No	1	
Yes	0.92 (0.82–1.03)	0.165
Beta‐blockers		
No	1	
Yes	0.58 (0.52–0.64)	<0.001
ACEI/ARB		
No	1	
Yes	0.83 (0.73–0.93)	0.001
Aldosterone blockers		
No	1	
Yes	0.81 (0.72–0.91)	<0.001
Inotropes		
No	1	
Yes	1.86 (1.65–2.10)	<0.001
IV diuretics		
No	1	
Yes	1.31 (1.15–1.50)	<0.001
Years of schooling		
0–4 years	1	
4–8 years	0.77 (0.67–0.88)	0.004
9–12 years	0.62 (0.54–0.71)	<0.001
≥13 years	0.63 (0.52–0.75)	<0.001
Dependant oedema		
No	1	
Yes	1.21 (1.05–1.39)	0.009
Atrial fibrillation/flutter		
No	1	
Yes	1.29 (1.11–1.51)	0.001
Mitral regurgitation		
No	1	
Mild	1.10 (0.95–1.26)	0.196
Moderate	1.18 (1.01–1.38)	0.038
Severe	1.18 (0.97–1.42)	0.091
Broad QRS		
No	1	
Yes	1.40 (1.26–1.56)	<0.001
Dilated cardiomyopathy		
No	1	
Yes	0.79 (0.69–0.91)	<0.001
Heart rate, 10 beats/min increase	1.02 (0.99–1.04)	0.130
Haemoglobin, 1 g/dL increase	0.95 (0.93–0.97)	<0.001
Serum sodium < 125 mEq/L		
No	1	
Yes	1.61 (1.30–1.98)	<0.001
Serum potassium > 5.5 mEq/L		
No	1	
Yes	1.04 (0.86–1.26)	0.708
Respiratory support		
No support	1	
Non‐invasive ventilation	1.27 (1.11–1.41)	<0.001
Invasive ventilation	5.04 (4.31–5.89)	<0.001

ACEI, angiotensin‐converting enzyme inhibitors; BMI, body mass index; CI, confidence interval; COPD, chronic obstructive pulmonary disease; HF, heart failure; HFmrEF, heart failure with mildly reduced ejection fraction; HFpEF, heart failure with preserved ejection fraction; HFrEF, heart failure with reduced ejection fraction; IV, intravenous; NYHA, New York Heart Association.

a Adjusted HR.

Patients with NYHA Classes III and IV at the time of admission had 36% [HR = 1.36; 95% confidence interval (CI): 1.16–1.60] and 61% (HR = 1.61; 95% CI: 1.37–1.90) higher mortality than patients with NYHA Class II. Patients with HFpEF had 23% (HR = 0.77; 95% CI: 0.64–0.92) lower 90 day mortality as compared with patients with HFrEF. Each unit increase in systolic blood pressure in millimetres of mercury was associated with 1% lower 90 day mortality (HR = 0.99; 95% CI: 0.98–0.99). Similarly, each unit increase in serum creatinine in milligrammes per decilitre was associated with 9% higher 90 day mortality (HR = 1.09; 95% CI: 1.06–1.12).

Individuals with more than 13 years of schooling reported 37% (HR = 0.63; 95% CI: 0.52–0.75) lower 90 day mortality as compared with individuals with <4 years of schooling. The presence of dependent oedema and atrial fibrillation was associated with 21% and 29% higher mortality as compared with individuals without such clinical signs at the time of admission. Broad QRS in electrocardiogram was associated with 40% (HR = 1.40; 95% CI: 1.26–1.56) higher mortality. Each unit increase in haemoglobin in grams per decilitre was associated with 5% lower 90 day mortality (HR = 0.95; 95% CI: 0.93–0.97). Lower serum sodium below 125 mg/dL at the time of admission was associated with 61% higher mortality (HR = 1.61; 95% CI: 1.30–1.98) as compared with individuals with serum sodium above 125 mg/dL. The requirement of both non‐invasive and invasive ventilation was associated with higher mortality as compared with individuals without the requirement of respiratory support.

## Discussion

Our findings based on the NHFR, the largest HF registry from India with adequate representation of all regions, provide crucial information on patient characteristics, treatment practices, and 90 day mortality outcomes of HF patients. In our cohort of relatively young patients with a mean age of 60 years, ischaemic heart disease was the predominant disease aetiology in three of four ADHF patients. Approximately one of two patients with HFrEF received GDMT. One of seven patients died during the first 90 days of follow‐up from the date of admission. Educational class, dependent oedema, QRS > 120 ms, atrial fibrillation, mitral regurgitation, haemoglobin levels, and serum sodium at baseline predicted 90 day mortality over and above the traditional risk score variables.

The hospitals included in the NHFR were spread across different regions of India with adequate coverage to the northern, southern, western, and eastern parts of India. Further, it covers states in all regions based on the epidemiological transition levels.^19^ The educational level of participants in the NHFR also reflects the national average educational level in this age group. As noted in several other registries,^6^, ^9^, ^10^ HF patients in India are younger than HF patients from high‐income countries.^3^, ^20^ Further, the mean age was a decade younger than the patients in the heart failure registry of patient outcomes^21^ study from China.

Male preponderance in HF incident cases as noted in the NHFR is a common observation in patients from India.^6^, ^9^ However, patients from HF registries in high‐income regions show a relatively high proportion of women.^3^, ^20^ High incidence of ischaemic heart disease in men may be one of the reasons for male preponderance as it is the commonest disease aetiology in the NHFR. However, a lack of tertiary care access for women in India has been reported earlier and may be responsible for the missing female HF patients.^22^

Heart failure equally affects the low and high socio‐economic strata in India. For example, one of four HF patients in the NHFR reported either no or less than 4 years of formal schooling. It is also striking that the 90 day HF mortality is highest in individuals with low educational status with a clear inverse relationship between educational level and mortality. A similar association has been noted in acute coronary syndrome registries^23^ and several other studies in India.^24^, ^25^, ^26^ Surprisingly, this inverse relationship persists in the NHFR even after adjustment for traditional factors associated with mortality including GDMT. A relatively high mortality in women, especially in the group who did not receive medications as per GDMT is also a cause of concern. The socio‐economic differentials in HF mortality in India need to be further investigated to provide clear recommendations for management in this high‐risk group of individuals.

In our registry, almost one of two patients with HFrEF received GDMT. This is a significant improvement based on the rates reported from previous HF registries in India.^6^, ^10^, ^27^ The setting up of registries and dissemination of its results may itself improve adherence to GDMT. The prescription rate of GDMT in the NHFR is comparable to that in acute HF patients in the ESC HF Long‐Term Registry,^3^ which provides data from six different regions of Europe. The uptake of GDMT in various HF registries is summarized in Supporting Information, *Table* *S2*. It is also important to note that those who received the GDMT survived longer than those who did not receive it during the initial hospital stay. The benefits of GDMT accrued in HFrEF were also noted in HFmrEF. This is consistent with findings from other studies that show that the combinations of ACEI/ARB, beta‐blocker, and aldosterone blocker benefit HFmrEF patients with a reduced mortality.^28^ Consistent with previous findings from India, the use of ARNI and devices like CRT and ICD was very low in the NHFR. The device treatment rate in the NHFR is at least nine times lower than that reported in high‐income countries,^29^ but similar to recent data from Tunisia.^30^ Issues related to affordability and accessibility to such treatment could be a major barrier in improving the use of such expensive but lifesaving treatment options in the Indian context.^31^

One of seven patients in the NHFR died during the first 90 days of follow‐up with an in‐hospital mortality of one in 15 patients. In‐hospital mortality rate in the NHFR was almost two‐fold higher than that in the ‘ESC HF Long‐Term Registry’ of acute HF patients.^32^ However, both in‐hospital and 90 day mortality rates in NHFR were lower than that reported previously in HF registries from India.^6^ Wider knowledge dissemination of benefits of the GDMT and its uptake may have improved the overall survival of HF patients in the NHFR. This is also consistent with the observation on relatively low mortality in HF patients who received GDMT in the NHFR. However, the mortality rate in registries from high‐income regions is even lower than that reported in the NHFR.^33^ It suggests that there is further scope for improvement and calls for innovative methods to improve the uptake of GDMT in eligible patients in India to reduce the mortality associated with HF.

### Strengths and limitations

National representation and data from all regions of India are the key strengths of our study. A uniform study protocol was followed at all sites for data collection with central monitoring for possible data errors. The lack of central adjudication committee to confirm the causes of death and type of re‐hospitalization is a major limitation of our study, and therefore, we restricted our analysis to all‐cause mortality. The registry included only patients from tertiary hospitals and departments headed by cardiologists, and the extent to which the findings from this study can be generalized to patients admitted in other hospitals with limited facilities is unclear. The new additions to GDMT such as the sodium‐glucose co‐transporter 2 inhibitors were not approved at the time of enrolment in the NHFR.

## Conclusions

Ischaemic heart disease is the leading cause of HF in India in a younger cohort of patients from the NHFR. Almost one of two eligible patients received guideline‐directed therapy in the NHFR. GDMT is associated with improved survival of HFrEF and HFmrEF. The 90 day mortality rate of one in seven patients and the sub‐optimal uptake of GDMT call for nationwide quality improvement initiatives.

## Conflict of interest

None declared.

## Funding

The Indian Council of Medical Research, New Delhi, supported this study financially (grant no. 50/1(9)/TF‐CVD/17‐NCD‐II). Panniyammakal Jeemon is supported by a senior clinical fellowship from the DBT‐Wellcome Trust India Alliance (IA/CPHS/20/1/505229).

## Supporting information


**Table S1:** Mortality outcomes.
**Table S2:** Prescription of disease modifying agents in heart failure.
**Figure S1:** Study sites in the NHFR.
**Figure S2:** Heart failure sub‐groups and 90‐day mortality.

**Appendix S1.** Supporting Information.
