# Supplementary material for: Clinical profile and 90 day outcomes of 10 851 heart failure patients across India: National Heart Failure Registry
Source: ESC Heart Fail. 2022 Aug 10;9(6):3898–908. doi: 10.1002/ehf2.14096 (PMC9773752; doi:10.1002/ehf2.14096)
Supplement: Supplementary file 1 — Table S1: Mortality outcomes. Table S2: Prescription of disease modifying agents in heart failure. Figure S1: Study sites in the NHFR. Figure S2: Heart failure sub‐groups and 90‐day mortality. [file EHF2-9-3898-s001.docx]

**Online supplement**

**Clinical presentation, management and 90-day outcomes of 10851 acute decompensated heart failure patients across India: Findings from the National Heart Failure Registry.**

Harikrishnan S^1^, Ajay Bahl^2^, Ambuj Roy ^3^, Animesh Mishra^4^, Jayesh Prajapati^5^, Manjunath CN^6^, Rishi Sethi^7^, Santanu Guha^8^, Santhosh Satheesh^9^, RS Dhaliwal^10^, Meenakshi Sarma^10^, Sanjay Ganapathy^11^, Panniyammakal Jeemon^12^ for the NHFR investigators^13^.

1. Professor of Cardiology, Sree Chitra Tirunal Institute for Medical Sciences and Technology, (SCTIMST), Trivandrum. drharikrishnan@outlook.com
2. Professor of Cardiology, Postgraduate Institute of Medical Education and Research (PGIMER), Chandigarh.
3. Professor of Cardiology, All India Institute of Medical Sciences (AIIMS), New Delhi.
4. Professor of Cardiology, North Eastern Indira Gandhi Regional Institute of Health and Medical Sciences (NEIGRIHMS), Shillong.
5. Professor of Cardiology, UN Mehta Institute of Cardiology and Research Centre (UNMICRC), Ahmedabad.
6. Professor of Cardiology, Sri Jayadeva Institute of Cardiovascular Sciences and Research (SJICR), Bangalore.
7. Professor of Cardiology, King George's Medical University (KGMU), Lucknow.
8. Professor of Cardiology, Medical College Hospital (MCH), Kolkata.
9. Professor of Cardiology, Jawaharlal Institute of Postgraduate Medical Education and Research (JIPMER), Pondicherry.
10. Scientist F, Division of Non Communicable Diseases, Indian Council of Medical Research (ICMR), New Delhi.
11. Additional Professor of Cardiology, Sree Chitra Tirunal Institute for Medical Sciences and Technology (SCTIMST), Trivandrum.
12. Associate Professor of Achutha Menon Centre for Health Science Studies, Sree Chitra Tirunal Institute for Medical Sciences and Technology (SCTIMST), Trivandrum.
13. The full list of NHFR investigators - please see appendix 1

Corresponding author:

Panniyammakal Jeemon

Associate Professor, Sree Chitra Tirunal Institute for Medical Sciences and Technology, (SCTIMST), Trivandrum, Kerala, India, 695011: [pjeemon@gmail.com](mailto:drharikrishnan@outlook.com)

Table S1: Mortality outcomes.

| **Variables** | **Overall (N=10851)** | **HFrEF (N=7082)** | **HFmrEF (N=2396)** | **HFpEF (N=1373)** |
| --- | --- | --- | --- | --- |
| Overall, 90-day mortality, n (%) | 1542(14.2) | 1107(15.7) | 264(11.0) | 171(12.5) |
| Overall mortality per 100 p-d, %  (95% CI) | 18.1(17.2 - 19.1) | 20.2(19.0 - 21.4) | 13.7(12.1 - 15.4) | 15.7(13.5 - 18.2) |
| In-hospital mortality, n (%) | 728(6.7) | 530(7.5) | 122(5.1) | 76(5.5) |
| **Men** |  |  |  |  |
| 90-day mortality, n (%) | 1040(13.9) | 800(15.7) | 152(9.2) | 88(12.3) |
| Mortality per 100 p-d, % (95% CI) | 17.7(16.6 - 18.8) | 20.2(18.9 - 21.7) | 11.2(9.7-13.1) | 15.3(12.4 - 18.9) |
| In-hospital mortality, n (%) | 471(6.3) | 369(7.2) | 64(3.9) | 38(5.3) |
| **Women** |  |  |  |  |
| 90-day mortality, n (%) | 502(14.9) | 307(15.6) | 112(15.2) | 83(12.7) |
| Mortality per 100 p-d, % (95% CI) | 19.2(17.5 - 20.9) | 20.1(17.9 - 22.5) | 19.5(16.2-23.4) | 16.1(13.0 - 19.9) |
| In-hospital mortality, n (%) | 257(7.6) | 161(8.2) | 58(7.9) | 38(5.8) |

HFrEF=heart failure with reduced ejection fraction, HFmrEF=heart failure with moderately reduced ejection fraction, HFpEF=heart failure with preserved ejection fraction, CI=confidence interval, p-d=person days.

**Table S2: Prescription of disease modifying agents in heart failure.**

| HF Registries | ACEI / ARB (%) | BB (%) | Aldosterone Blocker (%) | ARNI (%) |
| --- | --- | --- | --- | --- |
| NHFR (n=10851) | 58.3 | 75.9 | 65.5 | 3.6 |
| ESC-LT (n=4449) (Ref. 3) | 62-80 | 34-80 | 12-45 | - |
| ALARM-HF(n=3257)* | 79 | 50 | 32 | - |
| INTERCHF(Ref. 9) | 74 | 67 | 48 | - |
| INTERCHF(Range) | 48-85 | 48-85 | 27-59 | - |
| HERO (n=5620) (Ref. 21) | 43 | 50 | 70 | 0.8 |
| GWG (n=39982)** | 65 | 77 | 14 | - |
| First Euro Heart Failure Survey (n=10701)*** | 65 | 37 | - | - |
| HFrEF Subgroups | | | | |
| NHFR (n=7082) | 60 | 78 | 73 | 4.8 |
| HERO (n=668) (Ref. 21) | 50 | 61 | 84 | 3 |
| GWG (n=18398)*** | 72 | 84 | 20 | - |
| ALARM-HF (n=1298)* | 80 | 53 | 37 | - |
| COLES et al (n=1414)**** | 72 | 58 | 11 | - |
| Medanta (n=5590) (Ref. 10) | 66 | 82 | - | - |
| UK National Audit (n=74696)^#^ | 84 | 90 | - | - |

**Farmakis et al. Clin Res Cardiol. 2017 May;106(5):359-368, **Shah et al. J Am Coll Cardiol. 2017 ;70(20):2476-2486*

****Shoaib et al. Clinical Research in Cardiology (2019) 108:510–519, **** Coles et al. J Am Heart Assoc. 2015;4:e002303 doi: 10.1161/JAHA.115.002303, .^#^* *https://www.nicor.org.uk/wp-content/uploads/2020/12/National-Heart-Failure-Audit-2020-FINAL.pdf*

Figure S1: Study sites in the NHFR


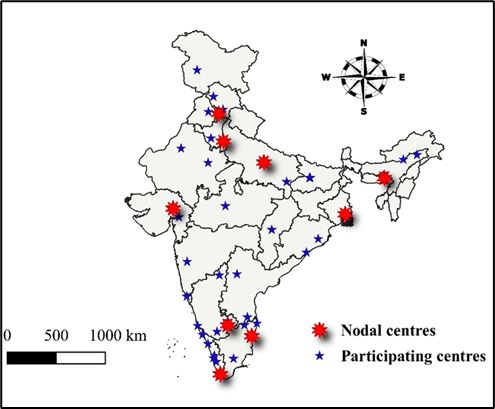


Fig S2. Heart failure sub-groups and 90-day mortality.


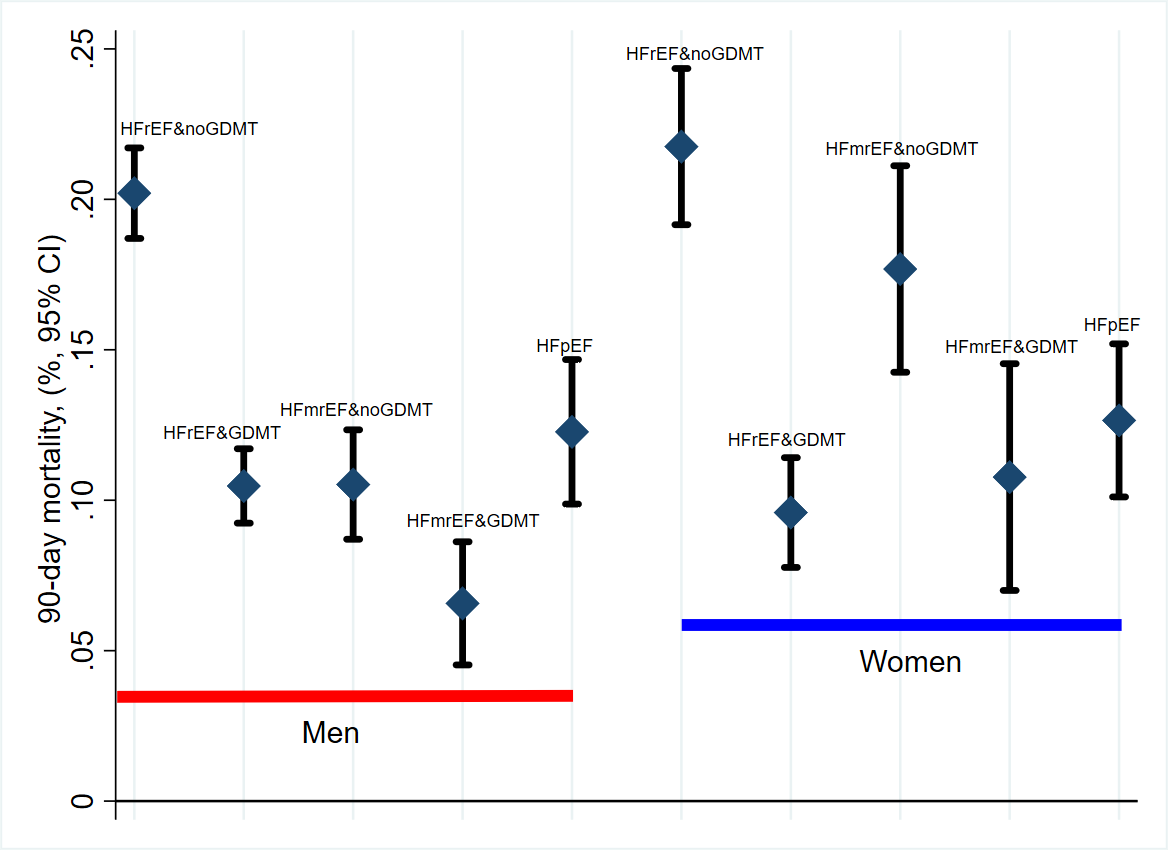


HFrEF=heart failure with reduced ejection fraction, HFmrEF=heart failure with moderately reduced ejection fraction, HFpEF=heart failure with preserved ejection fraction, GDMT=guideline directed medical treatment
